# Numerical Investigation of the Blast‐Induced Injuries Using an Open‐Source Detailed Human Model

**DOI:** 10.1002/cnm.3879

**Published:** 2024-10-21

**Authors:** Alberto Morena, Lorenzo Peroni, Martina Scapin

**Affiliations:** ^1^ Politecnico di Torino Turin Italy

**Keywords:** blast injury, blast wave, human model, numerical simulation, shockwave

## Abstract

Blasts are a threat both in military and civil contexts due not only to explosive devices but also to gas leakages or other accidents. Numerical models could aid to plan response strategies in the short and long term. Nevertheless, due to modeling complexities, a standardized computational framework has not been established yet. In this challenging context, the present study assesses the prediction of blast‐induced traumas by using the total human model for safety (THUMS) human model, which has never been attempted before to the authors knowledge. The pedestrian model is publicly available, hence the demonstration of its suitability to predict blast injuries could benefit the establishment of a common modeling framework. Therefore, the THUMS human model was exposed to different blast scenarios both in free field and partially confined spaces and the response of vital organs was investigated. Trauma patterns to internal organs of the THUMS were consistent with available experimental data and injury thresholds. In conclusion, THUMS open‐source human model demonstrated its validity to reproduce primary blast‐related injuries, addressing the development of standardization of numerical simulations of human response to explosions.

## Graphical abstract

The present study aims at validating the total human model for safety (THUMS) human model for predicting blast‐related injuries by simulating unconfined and partially confined blast scenarios. Simulations involved C‐4 generated blasts, comparing THUMS responses to experimental data and other models. Results showed THUMS ability to predict trauma to vital organs such as brain and lungs, supporting its use in simulations of human responses to explosions.

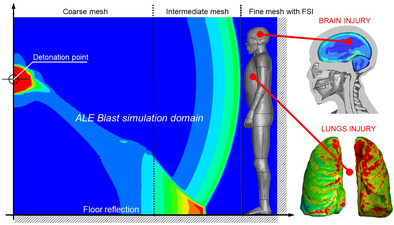

## 1 Introduction

Blast injuries on humans are a grave concern in modern society both in civilian and military contexts, particularly in areas affected by conflicts and terrorism.

The energy generated by an explosion can cause devastating physical trauma, affecting multiple body systems and resulting in severe injuries or even fatalities. The knowledge of mechanics and biomechanics of blast events is crucial for developing effective preventive measures, optimizing response strategies, and improving medical treatment for victims.

Furthermore, most public facilities are not designed with the purpose of minimizing the damages induced by improvised explosive devices (IED), while recent terrorist attacks have shown the vulnerabilities of these spaces [1].

The study of blast injuries involves an interdisciplinary approach that combines various engineering fields, such as fluid mechanics, structural mechanics, biomechanics, materials science, and medicine fields such as human anatomy and physiology.

Some frameworks brought together researchers and experts in these fields with the aim to work collaboratively to investigate the complex dynamics of blast events and their impact on human bodies [2]. Nevertheless, due to the complexity of the phenomena involved, human blast injuries are not yet completely understood, hence the development of a validated numerical procedure could enhance the knowledge on the matter.

In the realm of terrorism response, simulations enable emergency management teams to plan and prepare for potential blast incidents. By modeling different scenarios and assessing the effects of explosions on human populations and infrastructure, response strategies can be optimized, evacuation plans can be developed, and medical resources can be allocated more effectively.

In industrial settings, where explosions can occur due to accidents or hazardous materials, blast injury simulations contribute to improving workplace safety. By analyzing the potential consequences of explosions and identifying vulnerable areas or equipment, preventive measures can be implemented, safety protocols can be enhanced, and risk assessments can be conducted to minimize the impact on human lives and property.

In the military context, blast injury simulations aid in the design of protective gear and vehicles, allowing soldiers to withstand explosions with reduced risk of injury. These simulations also help in developing blast‐resistant structures, such as fortified buildings and vehicles, to safeguard military personnel and critical assets [3, 4].

From a mechanical perspective, the analysis of blast injuries focuses on the study of blast waves and their interaction with the human body. A blast wave is a high‐pressure shock wave that propagates rapidly through a medium following an explosion. It consists of a compression wave followed by a rarefaction wave, creating a sudden increase and decrease in pressure. This abrupt pressure change exerts immense forces on the human body, leading to various types of injuries.

The biomechanical aspect of blast injuries involves understanding the response of human tissues and organs to the blast wave. The human body is a complex system with different materials and structures, each with its own mechanical properties and vulnerabilities. When exposed to a blast wave, the body experiences dynamic loading and stress, causing tissue damage, organ rupture, bone fractures, and other traumatic injuries.

Indeed, extensive literature has been developed on blast‐related injuries concerning both blunt and penetrating damages [5, 6]. Nevertheless the traumas are generated by complex wave reflection patterns inside the human body and, in the case of mild traumas, the damages could be difficult to observe immediately, but could occur at later times.

The blast‐related injuries are generally divided into primary, secondary, tertiary, quaternary, and quinary. The primary injuries are due to the impingement of the blast wave on the surfaces of the body, which generates pressure waves inside the tissues. The secondary injuries are caused by the projection of debris following the blast winds, which eventually collide with the human tissues. Tertiary injuries are mainly caused by the displacement of the body due to the explosion, which might foster its collision with surrounding objects or structures. Quaternary and quinary blast injuries are related to the burns, toxic effects and other chemical and radioactive damages.

Blast‐induced traumatic brain injuries (TBI) are the main injury causes in military personnel [7]. Often, the TBIs occur as mild‐TBI, which are complex to diagnose and can cause various symptoms, such as headaches, depression, and sleep disorders [8].

Experimental tests are performed with specialized equipment, such as shock tubes or explosive setups, to generate controlled blast waves and assess their impact on humans.

Pioneering work from Bowen et al. [9] investigated human blast lethality through experimental tests on animals. More recently, Bass et al. collected and statistically analyzed more than 2550 experiments on animals for both short and long duration blasts and found similarities with Bowen's work [10, 11]. In the meantime, research on blast injuries also made use of postmortem human subjects (PMHS) and anthropomorphic test devices [12]. Bir [13] used instrumented PMHS heads to investigate TBI and collected useful data concerning intracranial pressure (ICP) and skull strain. The limitation of that study was due to the absence of the neck which caused the head–neck kinematics to be unrealistic in the long term. Hence, Iwaskiw et al. [14] used PMHS head–neck samples to collect pressure and displacement data during shock tube reproduced blasts. The shock tube setup was also used by Goeller et al. [15], but the head injury pattern was reproduced by means of a simplified head surrogate model, that is, a polymer‐made ellipsoid filled with a water‐like fluid to replicate cerebrospinal fluid (CSF) fluid. Merkle et al. [16] developed an anthropomorphic human surrogate torso to investigate internal organs blast injuries due to open field explosions and provided useful insights into the pressure wave propagation inside vital organs such as lungs, stomach and liver.

Indeed, experimental research is complex both in terms of costs and also for ethical reasons, hence the urge of validated numerical procedures to provide useful insights on the effects of blast on humans is evident.

Computational modeling enables the simulation of blast waves and their interaction with the human body, providing insights into the biomechanical response and injury patterns. These models need to incorporate detailed anatomical representations, material properties, and tissue behavior to accurately replicate the physical phenomena involved in blast injuries. Chafi et al. [17] developed a three‐dimensional finite element model of a human head and validated it against impact loadings on cadavers before using it to simulate blast events. Taylor et al. [18] built a more detailed head–neck model from an available dataset and subjected the resultant model to different blast scenarios. Following Taylor et al. work, Rezaei et al. [19] simulated primary blast injury on a head model using experimental data on partially confined explosions. Considering the importance of correctly evaluating brain traumatic blast injuries, also other researchers focused on developing their own computational head models for explosion studies [20, 21, 22, 23, 24, 25, 26].

Concerning thoracic injuries, Greer et al. built a bidimensional simplified slice model of the torso to simulate blast effects [27]. Similarly, in [28] slice torso models of human and sheep were subjected to blasts from different orientations. Successively, Goumtcha et al. [29] used a tridimensional only‐torso biomechanical model to investigate internal organs wave patterns when subjected to experimental scenarios by Merkle et al. [16]. Likewise, Ward et al. [30] used a human torso finite element model to replicate load blast cases starting from Bowen's lethality curves. The authors used the *load_blast card implemented in LS‐Dyna in order to achieve computational efficiency.

Since many vital organs are located in the torso, other noteworthy numerical studies on blast effects on the thorax were published [31, 32].

Numerical studies on blast loadings often involve customized human models developed by various research groups. As a result, these models are not readily accessible to the public.

In recent years, some human body models developed for crashworthiness analysis have been released to the public, such as the Toyota Total Human Model for Safety (THUMS) [33]. Furthermore, complete human body models such as the THUMS or the Global Human Body Modeling Consortium (GHBMC) [34] have reached a level of detail for which they could be used to reproduce blast loading conditions.

The aim of the present work is to investigate the possibility of using the THUMS pedestrian model to reliably reproduce blast traumas in free air and partially confined conditions. The THUMS model has been used to model underbody blast scenarios [35] and for only‐torso modeling [36], but to the best of the authors' knowledge no attempt has been made to evaluate THUMS ability to reproduce a whole body blast loading.

In this work, using LS‐Dyna software, Arbitrary Lagrangian Eulerian (ALE) fluid structure simulation procedure was used and its capability to produce reliable results was validated against experimental data for steel structures. Furthermore, impact validation cases used to calibrate THUMS human model are presented. Finally, the blast effects on THUMS were reproduced and the results were compared to data from the literature for free air explosions. In conclusion, the increase in damaging effects on humans due to the partial confinement were analyzed.

## 2 Methods

Finite element models are commonly used to simulate explosions since they are able to considerably reduce the costs and times with respect to experimental testing. Nowadays, empirical methods, such as the ConWep [37], are widely implemented in the commercial softwares, but most researchers and engineers often resort to computational fluid dynamics (CFD) when dealing with complicated geometries. In fact, the reflections with the walls of a structure could generate very complex patterns, which might be difficult to correctly predict with empirical models. For this reason, finite element softwares, like Ansys LS‐Dyna, have implemented several algorithms to describe explosions in a physics‐based manner, such as the ALE method with Fluid–Structure Interaction (FSI) algorithms.

The ALE approach has been used in several works to reproduce explosions, reporting accurate results [38, 39, 40, 41].

The ALE algorithm operates an automatic rezoning of the solution. As a matter of fact, when ALE is activated, the solver divides the solution in two parts, the Lagrangian timestep and the advection step. During the Lagrangian step the solver uses an explicit time integration scheme and the mesh deforms following the deformation of the material, while during the advection step the solution obtained at the Lagrangian step is remapped on a less distorted mesh. The advection step can be implemented in order to remap the solution on the initial undeformed mesh at each timestep, in this way the approach is Eulerian. In the software LS‐Dyna different advection schemes are implemented. Among all, a modified donor cell with Half Index Shift is usually adopted to preserve the energy balance during explosion simulation [42, 43].

Following the two‐step approach, the Navier–Stokes equations are integrated in time [42, 44].

Generally, ALE blast simulations are performed with a multi‐material mesh, which means that the same mesh is used both to describe the explosive and the fluid in which the shockwave will propagate.

Indeed, since the fluid will require a great amount of finite elements, ALE simulations are computationally more demanding than pure Lagrangian simulations. For this reason, the multi‐material mesh is typically formed by under‐integrated elements, hence an hourglass control is needed to avoid unrealistic deformations.

As already mentioned, both the air and the explosive require a material model and an equation of state to correctly determine their behavior.

The air is usually modeled as a material with no capability to resist shear stresses, hence with a *MAT_NULL in LS‐Dyna, and with an EOS of an ideal gas with isentropic coefficient γ=1.4, thus a *EOS_LINEAR_POLYNOMIAL, which has the following form:

(1) $$P=C_{0}+C_{1}\mu +C_{2}\mu^{2}+C_{3}\mu^{3}+E\left(C_{4}+C_{5}\mu +C_{6}\mu^{2}\right)$$

where the *C* coefficients are the coefficients of the polynomial, $\mu =\rho /\rho_{0}-1$ where $\rho /\rho_{0}$ is the ratio between the current density and the initial density, and *E* is the internal energy. For an ideal gas treatment of air all the *C* coefficients are set equal to zero except for $C_{4}=C_{5}=\gamma -1=0.4$.

For the explosive, the detonation is generally defined by a Jones–Wilkins–Lee EOS, which links the relative volume and the explosive energy to the developed pressure as follows:

(2) $$P=A\left(1-\frac{\omega }{R_{1}V}\right)e^{-R_{1}V}+B\left(1-\frac{\omega }{R_{2}V}\right)e^{-R_{2}V}+\omega \frac{E}{V}$$

where *V* is the relative volume, *E* is the energy of detonation, and *A*, *B*, *R*_1_, *R*_2_, and ω are coefficients specific for the explosive.

Furthermore, high explosives need a material model to characterize the chemical energy release. Typically, the material model used for explosives in LS‐Dyna is *MAT_HIGH_EXPLOSIVE_BURN where the detonation velocity and Chapman–Jouget pressure of the modeled explosive are required as input.

The material models and equation of state parameters for the air and trinitrotoluene (TNT) and C‐4 composition explosives are reported in Table 1.

**TABLE 1:** Material model and equation of state parameters for air, TNT, and C‐4 [38, 45, 46].

Material	$C_{1}\;\left[-\right]$	$C_{2}\;\left[-\right]$	$C_{3}\;\left[-\right]$	$C_{4}\;\left[-\right]$	$C_{5}\;\left[-\right]$	$C_{6}\;\left[-\right]$	$C_{7}\;\left[-\right]$	$E_{0}\;\left[\mathrm{MPa}\right]$	$V_{0}\;\left[-\right]$	$\rho_{0}\;\left[\frac{\mathrm{ton}}{mm^{3}}\right]$
Air	0	0	0	0.4	0.4	0	0	0.25	1	1.293e − 12
Material	$A\;\left[\mathrm{MPa}\right]$	$B\;\left[\mathrm{MPa}\right]$	$R_{1}\;\left[-\right]$	$R_{2}\;\left[-\right]$	$\omega \;\left[-\right]$	$E_{0}\;\left[\mathrm{MPa}\right]$	$V_{0}\;\left[-\right]$	$\rho_{0}\;\left[\frac{\mathrm{ton}}{mm^{3}}\right]$	$D\;\left[\frac{\mathrm{mm}}{s}\right]$	$P_{\mathrm{CJ}}\;\left[\mathrm{MPa}\right]$
TNT	3.712e5	3231	4.15	0.95	0.3	7000	1	1.59e‐9	6.93e6	2.1e4
C‐4	6.0997e5	1.295e4	4.5	1.4	0.25	9000	1	1.601	8.193e6	2.8e4

## 3 Modeling of Blast Loading Effects on the THUMS

In the present work the blast scenario of a 50th percentile male standing at 2.3 m from an explosive charge standing at the level of its sternum has been investigated. Hence the THUMS pedestrian human model was subjected to two levels of blast exposure: the detonation of a 2.3 kg Composition‐4 charge and the detonation of a 0.9 kg Composition‐4 charge. Altogether three blast scenarios were taken into account: the detonation of 2.3 kg of C‐4 in open field, the detonation of 0.9 kg of C‐4 in open field and, finally, the blast exposure due to 2.3 kg of C‐4 detonating in a partially confined environment.

The first case generates at the location in which the THUMS is standing a condition in the proximity of Bowen's curve for lung blast injury [9]. In the literature, no experimental cases on PMHS for a similar explosion situation are present. However, Tan et al. [47] developed a standing FEM human model specifically for blast studies and exposed the virtual surrogate to an identical blast. In [47], the traumatic brain response is analyzed and pressure histories are reported. In the present work the pressure patterns simulated by Tan et al. are taken as reference to determine if the THUMS pedestrian model can cope with virtual surrogates specifically developed for blast analysis.

Unfortunately, in the work by Tan et al. [47] the response of vital organs such as lungs and liver is not reported. Hence, in the present study the second blast scenario of an explosion of 0.9 kg of C‐4 was simulated in order to replicate the experimental setup of Merkle et al. [16]. In [16], a human surrogate only‐torso dummy was subjected to the detonation of different C‐4 charges and the pressure developed in lungs, liver, and stomach was acquired by means of pressure sensors. The comparison between data coming from virtual sensors in the internal organs of THUMS and from sensors of the experimental torso surrogate will determine THUMS thoracic and abdominal biofidelity.

Finally, a partially confined blast simulation will be analyzed to evaluate the injury increase due to the confinement.

The simulation of the 2.3 kg C‐4 explosion has been performed both by means of the LBE method and the ALE method, in order to evaluate whether the former technique is able to produce reasonable results in a faster way. The usage of the ALE method is strongly advised when dealing with complex geometries such as a human body model, since the empirical abacuses are not able to take into account the fluid dynamics of the blast wave travelling around the body. Nevertheless, given the advantage in terms of computational time, a less accurate LBE simulation could also be sufficient for the scope.

**FIGURE 1:**
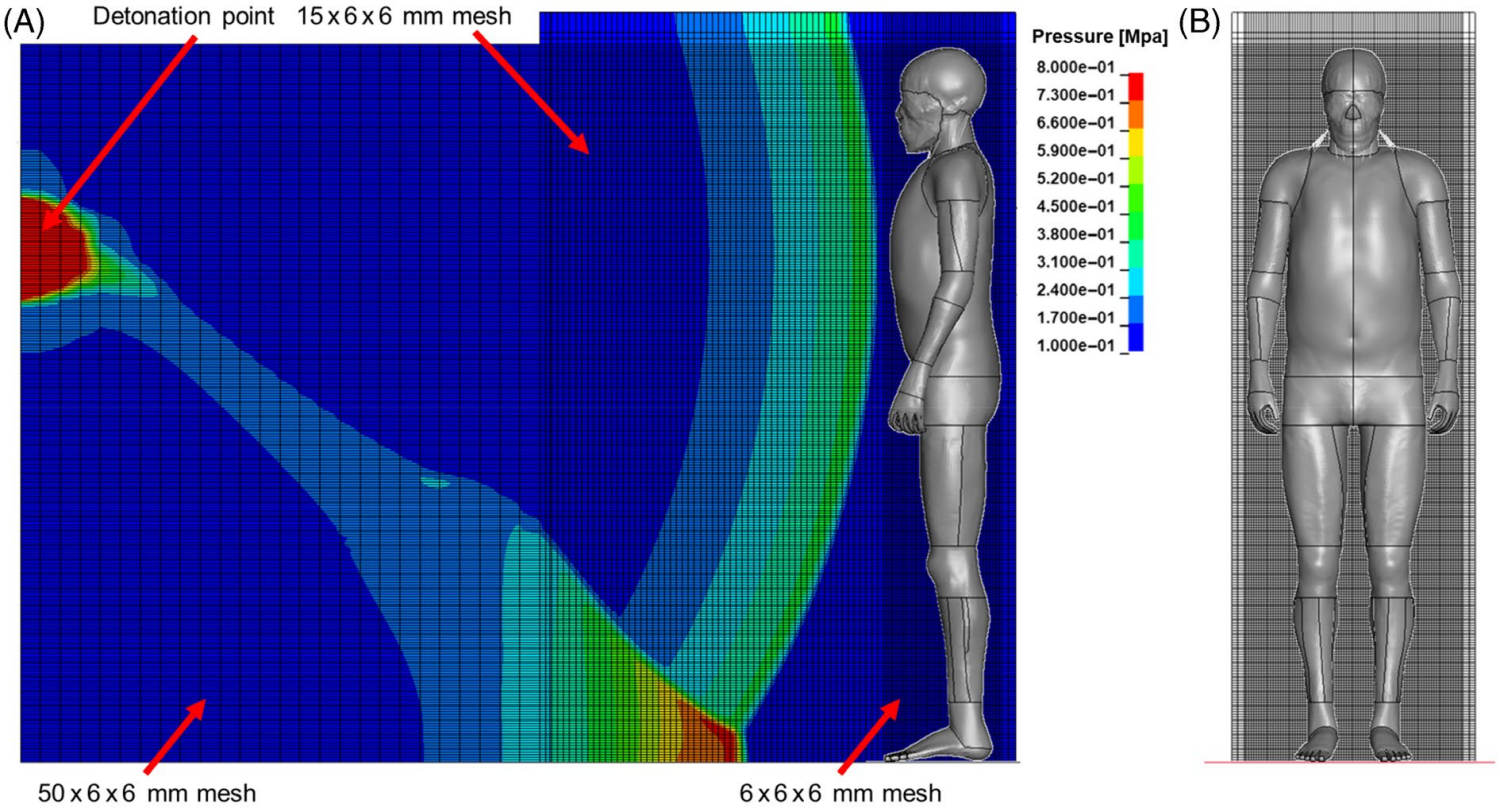
3D ALE model immediately after the remeshing of the 2D axisymmetric detonation simulation (i.e., starting time of 3D simulation). (A) Lateral plane view. The ALE mesh was sectioned at the human model sagittal plane. The time is 1.69 ms after the 2.3 kg C‐4 detonation. (B) Front view.

All the ALE simulations were performed resorting to a 2D to 3D remapping procedure to reduce computational time. Hence, a 2D axisymmetric simulation of the first milliseconds of the detonation and ground reflection was built, then the results were mapped in a 3D geometry with the THUMS model. A section view of the 3D ALE model with the remapped pressures from the 2D axisymmetric simulation of the 2.3 kg C‐4 detonation is reported in Figure 1. The 3D model extends up to the detonation point with the aim to correctly describe the reflected wave and the Mach stem formation. The 3D ALE mesh was built with a higher density around the THUMS model to have the ALE elements with the same dimension of the THUMS skin mesh, in order to avoid inaccuracies during the fluid structure interaction computations. On the other side, to reduce computational time a coarser ALE mesh was used far from the THUMS. Hence the ALE mesh had 6 × 6 × 6 mm elements around the human model, with a first transition zone to 15 × 6 × 6 mm and finally to 50 × 6 × 6 mm. The 15 mm mesh is the one into which the 2D solution is remapped, thusly it was chosen to avoid too large mesh ratio during the remapping from the mesh used for the 2D axisymmetric analysis which was of 5 × 5 mm, which would have caused an unrealistic smoothing of the pressure wave in air. Nonetheless, it was assured that in the transition between the 15 mm and the 6 mm mesh no numerical disturbance of the pressure wave happens. The 50 mm mesh was used for the zone in which the wave reflected from the ground has a low‐pressure intensity at the time of remapping, after checking that this wide mesh does not affect negatively the results in the THUMS region.

Furthermore, the ALE mesh extensions in the vertical, that is, above the head, and in the coronal plane of the human model were of 30 mm larger than the extremities of the THUMS in both directions, resulting in a negligible influence of the domain borders (Figure 1b).

The whole ALE mesh was subjected to a significant analysis to verify that a finer mesh would not provide better results and that the transition between the mesh regions would not introduce numerical errors. Finally, the ALE mesh counted almost 4 million elements.

## 4 Results

Setting up numerical models often requires comparison with experimental data to validate the simulations. In this section, experimental data from the literature are reproduced by means of numerical simulation with the aim of properly calibrating the simulations.

Confined explosion experiments from Yao et al. [48] are used to validate the predicted deformation of structures under blast loading. Successively, the THUMS model validation cases relevant for the present study are presented and discussed. Finally, the results of the blast cases involving the THUMS human models are displayed.

### 4.1 Deformable Structure Validation

For the purpose of validating the blast simulation procedure which has been used in the present work, the experimental tests performed by Yao et al. [48] were taken into consideration. During the aforementioned experimental campaign, three steel boxes with scaled geometries were subjected to scaled blast loadings with the aim of obtaining scaling criteria to predict structural effects of confined explosions. The smallest chamber had square walls measuring 300 mm of edge length and was subjected to explosions of TNT charges ranging from around 13 to 40 g, while the largest had 600 mm edge length walls and was tested with TNT charges from almost 100 to 337 g [48]. The experimental tests provided useful insights on the final deflection of the steel boxes tested. In [49], the deformation history of the center plate of the middle chamber (450 mm edge length) exposed to 84 g of TNT is reported. The deformation was measured by means of DIC technique with two high‐speed cameras [49].

In this paper the confined blast inside the middle box (SB‐II) was reproduced by means of S‐ALE solver.

The S‐ALE mesh used in the simulation was of 5 mm and the same dimension was used for the Lagrangian mesh describing the steel box.

In addition, in this case the symmetry of the setup was exploited, hence only a quarter of the steel box was modeled.

The box was made of Q235 steel which can be described by a Johnson–Cook material model with the parameters reported in Table 2 [48, 49].

**TABLE 2:** Johnson–Cook parameters for Q235 steel from [48, 49].

$\rho \;\left[\mathrm{kg}/m^{3}\right]$	$E\;\left[\mathrm{GPa}\right]$	$G\;\left[\mathrm{GPa}\right]$	$\nu \;\left[-\right]$	$A\;\left[\mathrm{GPa}\right]$	$B\;\left[\mathrm{GPa}\right]$	$n\;\left[-\right]$	$c\;\left[-\right]$
7.8e3	210	80.8	0.3	370	438	0.6	0.01

The ratio between the mass of the TNT charge and the volume of the chamber is 0.39 kg/m^3^ which is higher than the limit for full afterburning, thus there is not enough oxygen to fully react with the detonation products and for this reason no afterburning effects were included.

**FIGURE 2:**
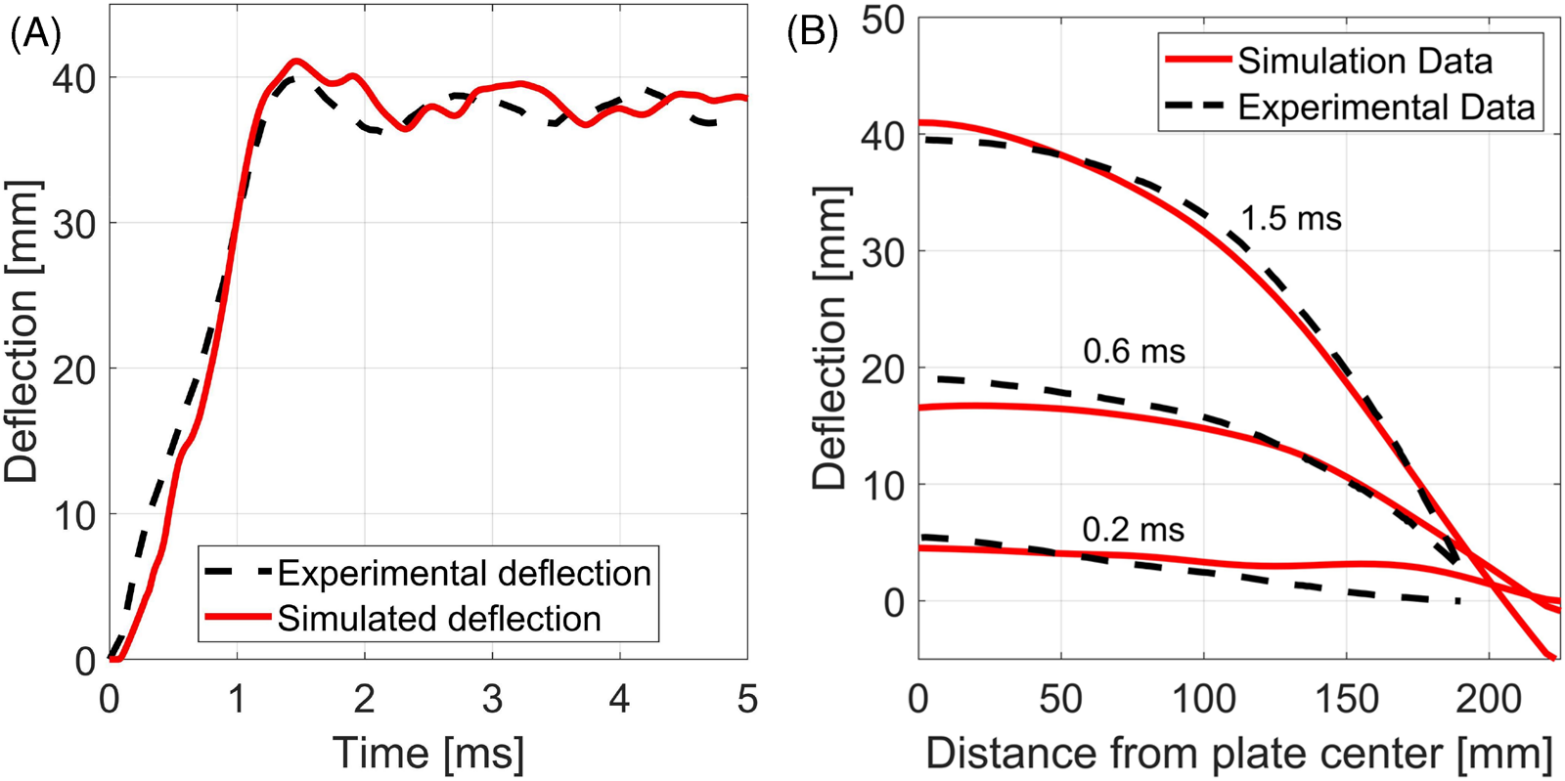
Comparison between experimental deflection measured by DIC [49] and deflection from S‐ALE simulation: (A) deflection history for the center of the plate; (B) deflection of the midplane of one of the walls at times 0.2, 0.6, and 1.5 ms from detonation.

In Figure 2a the experimental DIC deflection of the center of one of the walls is plotted with the results from the S‐ALE simulation. As it can be seen, the numerical model is able to correctly predict the peak displacement of the steel plate. Furthermore, also the deflection of the midplane of one of the walls predicted by the numerical simulation developed in this work was compared with the experimental DIC data and the accuracy of the model is appreciable in Figure 2b.

With the aim to check the coherence of the deformations of a wider steel box a numerical model was built with a chamber of the same geometry but scaled by a factor 10 with respect to the box SB‐I, resulting in a steel chamber with 3000 mm edges and 20 mm thickness of the plates.

The wider steel box was then subjected to three blast loads caused by cubic TNT charges with increasing mass. The input data and the results of the explosion simulations are reported in Table 3.

**TABLE 3:** Simulation data from the 3 m wide chamber.

*W* [kg]	*Z* [m/kg^⅓^]	δ [mm]	δ/H [−]
15.5	0.602	183	9.15
23.5	0.524	250	12.5
30.2	0.482	309	15.45

*Note: W* is the TNT charge mass, *Z* is the scaled distance, δ is the deflection of the center of the wall and δ/H is the deflection/thickness ratio.

In [48], a relationship between the δ/H deflection/thickness ratio and the scaled distance *Z* was proposed, which is reported in the following:

(3) $$\delta /H=3.098Z^{-2.12}$$

The scaled distance *Z* is the ratio between the distance from the explosive and the cubic root of the charge mass.

**FIGURE 3:**
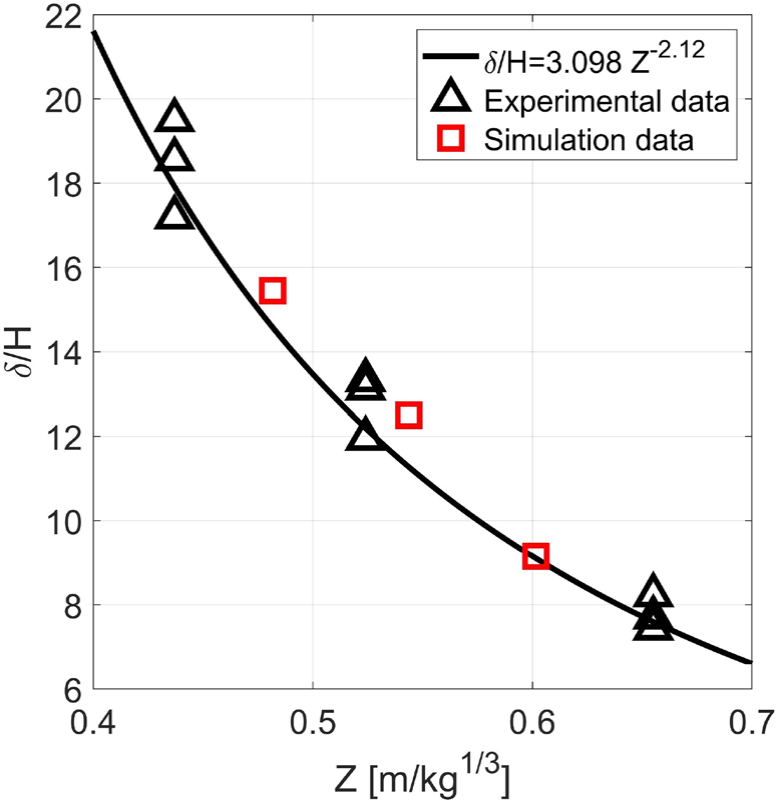
Deflection/thickness ratio on scaled distance Z plot representing: Experimental data from [48], Equation (3) and simulation data from the 10× scaled steel chamber.

In order to check if the wider scaled box is coherent with the experimental data from [48], the δ/H are plotted in Figure 3 along with the aforementioned experimental data and the fitting Equation (3).

From Figure 3, it is clear that the simulations of the detonation inside the 10× scaled chamber are coherent with the experimental data from [48] and the fitting Equation 3.

Indeed, as mentioned before, the ALE algorithm is computationally more expensive than a pure Lagrangian technique. In LS‐Dyna software the abacuses from ConWep can be invoked with the *LOAD_BLAST_ENHANCED (LBE) keyword [37]. The ConWep tables have been implemented for TNT spherical charges and free air explosions, which is not the case of the steel box. However, a “method of images” can be used which serves to describe the reflecting waves [50]. The LBE method of images is an approximated approach for confined blasts, which could provide a first esteem of blast effects in faster times, giving the possibility to test different scenarios.

**FIGURE 4:**
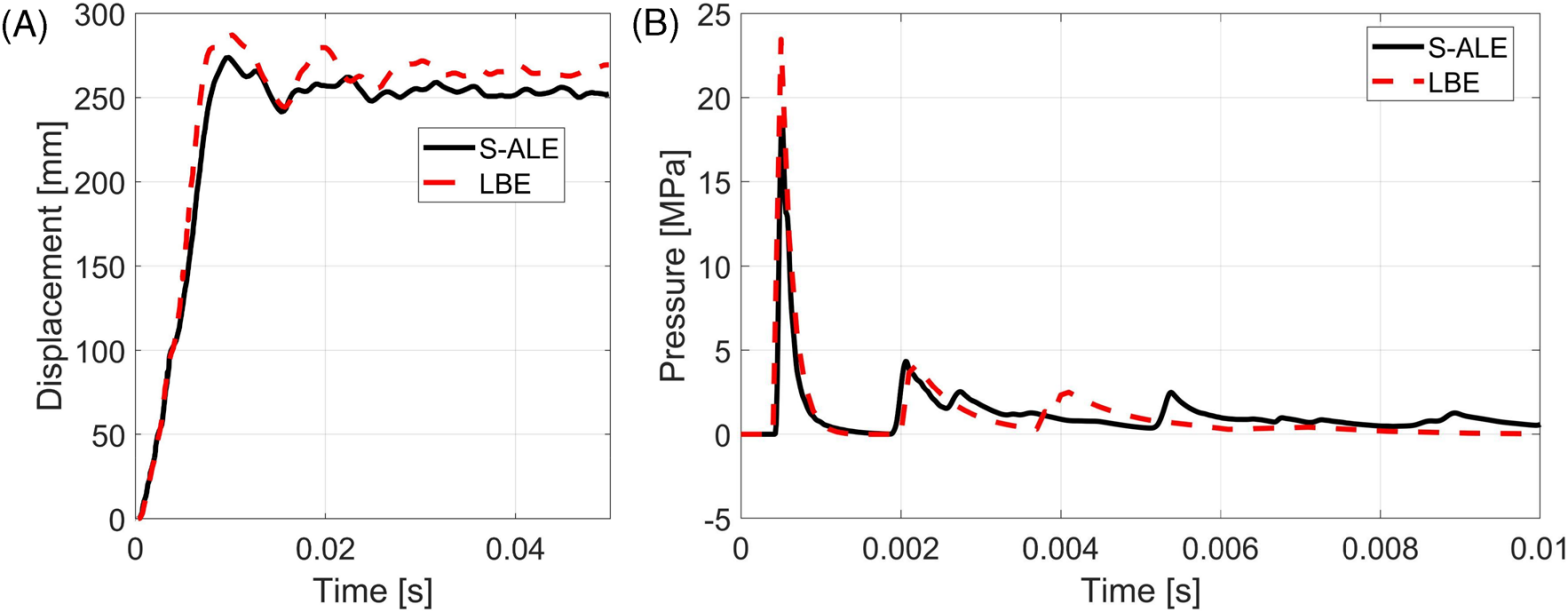
(A) Center plate displacement history for LBE (Method of Images) and S‐ALE simulations of the wider steel box; (B) pressure history of a sensor placed in the center of one of the walls for S‐ALE and LBE simulations.

Following the method of images, a simulation was performed in LS‐Dyna where the reflection of the mirror charges was developed up to the second order reflections. In Figure 4a the results in terms of displacement history of the center plate are reported for the LBE (Method of Images) with mirror charges and for the S‐ALE simulations. It can be highlighted that the difference in the maximum plate deflection between ALE and LBE simulations is less than 5%, hence the two methods appear to be coherent.

As a matter of fact a similar deformation history should be caused by the same loading history. To check that this statement applies to our case in Figure 4b the pressure histories acquired by the same sensor for the two cases are reported. Comparing the two pressure histories, it can be concluded that the LBE method of images is able to correctly reproduce the pressure peaks caused by the reflections of the waves.

It is important to highlight that the LBE method of images does not take into account the venting hole on the ceiling of the steel box. However, this discrepancy seems not to avoid the LBE simulation to provide useful results.

Indeed the advantage in terms of computational time can not be underestimated, since the two methods were run on the same Dell PowerEdge R360 with 40 Intel (R) Xeon (R) E5‐2640 v4 CPUs and the ALE simulation required 6 h and 30 min, while the LBE simulation required only 8 min. It is clear that for a first approximation simulation the LBE method of images can be a useful technique.

### 4.2 THUMS Validation

Simulating the human response to blast scenarios implies that the biofidelity of the applied models is correctly represented. Thus, it is crucial to understand the conditions in which the THUMS model was calibrated in order to assess the field of application of the FEM model.

The present section intends to present the validation tests through which the THUMS model was calibrated with a double purpose. Firstly, it provides proof that the THUMS model is able to reproduce experimental tests which have become a standard. Secondly, the range of pressure, strain, and strainrate reached during the tests are evaluated. These ranges will be compared with the solicitations imposed by the blast scenarios investigated in the next sections to check if the THUMS human model was used within its validation limits.

The results of the validation cases presented will be used as comparison with the blast simulation outcomes, considering that if the maximum pressure, strain, and strainrate obtained during blast loading will be within the limits of the validation setups, then the THUMS model is being used in the range of solicitation for which it was calibrated.

The THUMS pedestrian V4.02 developed by the Toyota Motor Corporation and Toyota Central R&D Labs., Inc. has been calibrated with extensive experimental data on cadavers [33]. The head pressure response to frontal impulsive impact loading was fine‐tuned thanks to the experimental tests from Nahum et al. [51] on cadavers. Furthermore, brain kinematics have been correlated to Hardy et al. [52] and Kleiven et al. [53] data on PMHS.

Many authors used the cited experimental campaigns to validate their human head surrogate for blast loading analysis [18, 54, 55], hence the material models and mechanical properties attributed to the different components of the head are often in great agreement with the THUMS [33]. The grey matter is modeled by means of a Kelvin‐Maxwell viscoelastic material model making its response dependent on the loading rate. The same material model is used for the CSF, indeed with different parameters with respect to grey matter. On the other hand, the skull is described with an elastic–plastic material model.

For the frontal impulsive impact loading case the pressure, strain, and strainrate obtained in the brain were investigated to identify the range of validity of the head model. Therefore, Nahum et al. [51] validation case was simulated with the THUMS head only model and the virtual sensors in the grey matter were analyzed.

**FIGURE 5:**
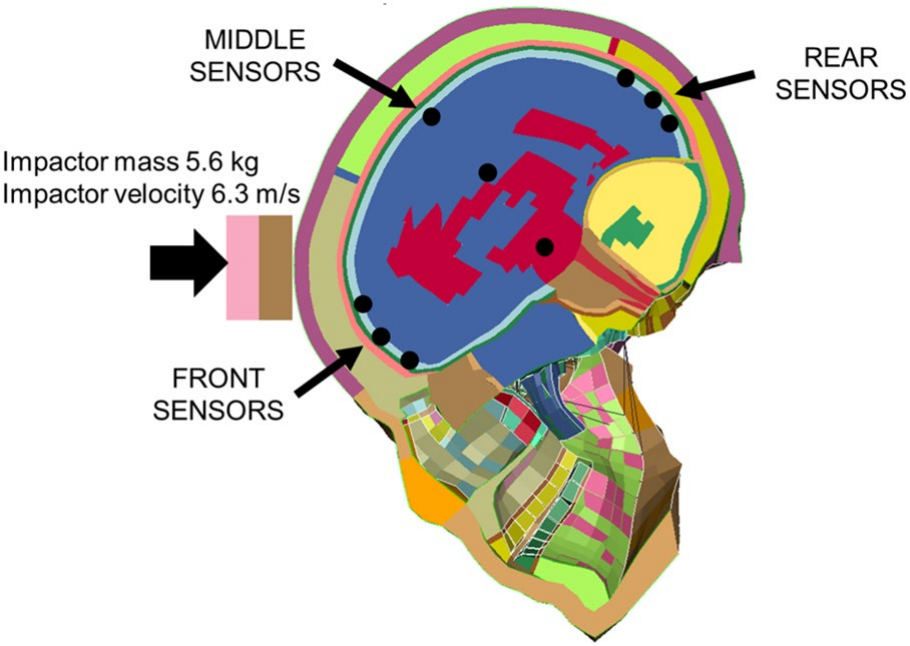
Simulation of the Nahum et al. experiment [51] by means of the THUMS head model with locations of the front, middle and rear sensors in the grey matter.

In Figure 5 the brain validation case setup is reported with the positions of the grey matter sensors. The impactor weighs 5.6 kg and has an initial velocity of 6.3 m/s toward the forehead.

The results from the simulation of the Nahum et al. test with the THUMS human model are reported in Table 4. For sake of conciseness the complete pressure histories of the sensors are not reported hereafter, since they can be found in the THUMS report [33].

**TABLE 4:** Results in terms of peak pressure, maximum strain, and maximum strainrate acquired by the front, middle and rear sensors during the head frontal impact simulation.

	Peak pressure [kPa]	Maximum strain [−]	Maximum strainrate [s^−1^]
Front	223	0.027	10
Middle	154	0.053	22
Rear	−50	−0.055	25

Indeed, also the thorax and abdomen of the THUMS have been validated through comparison with experimental data [33, 56, 57]. In the present work the biomechanical accuracy of lungs, liver, and stomach will be assessed. In the THUMS model the lungs are characterized by a fluid‐like material with the definition of a viscosity parameter which determines the evolution of the deviatoric stress with respect to deviatoric strainrate. Differently, liver and stomach are described with a force versus gauge length curve and a damping parameter properly calibrated for both the vital organs.

**FIGURE 6:**
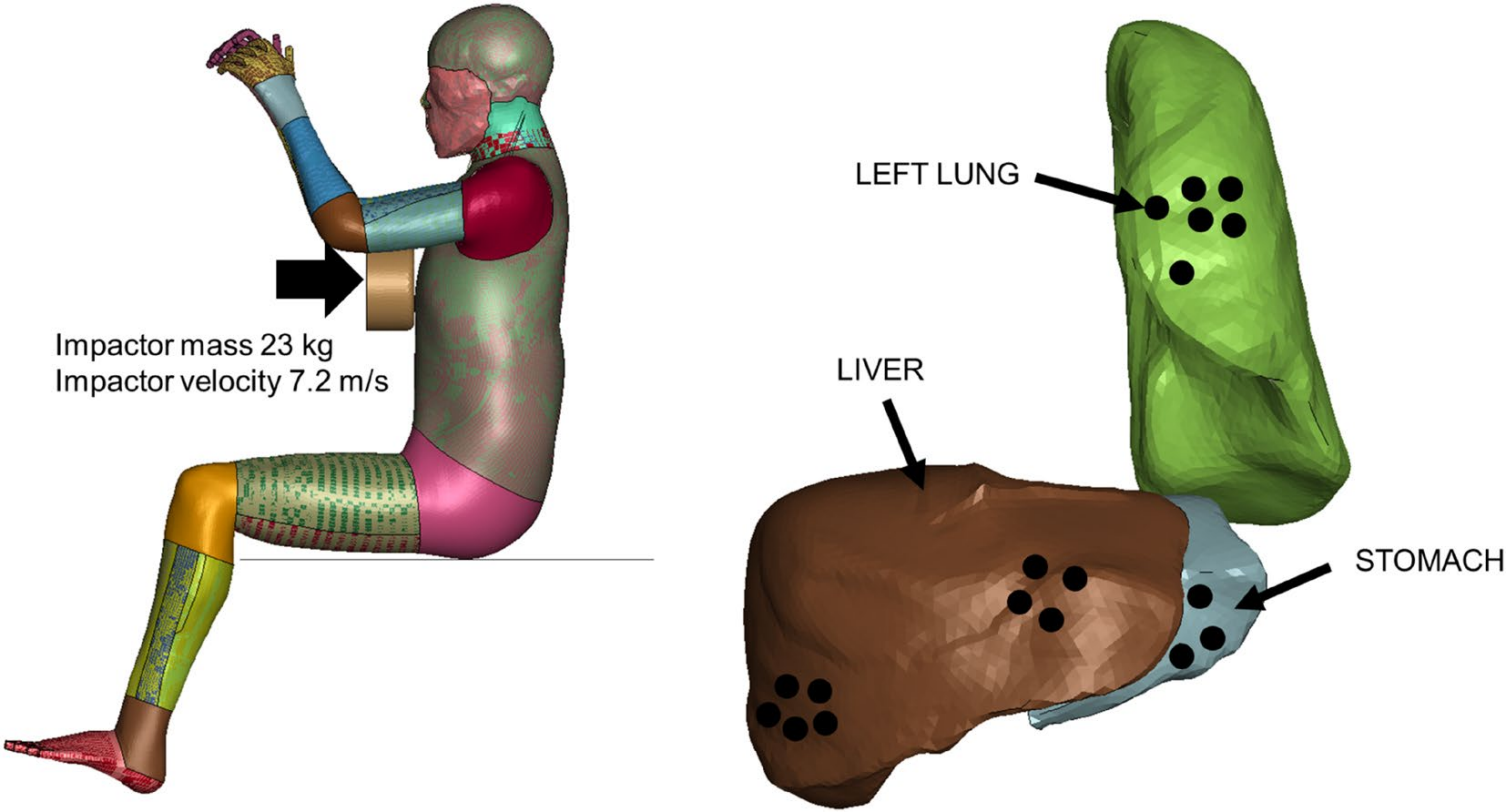
Simulation of the Kroell et al. experimental test [56] by means of the THUMS model (left) and location of the sensors for the left lung, liver and stomach (right).

The test by Kroell et al. [56] of an impactor hitting the anterior part of the thorax serves as a validation case for THUMS thoracic model. The simulation setup to reproduce the experimental test and the sensors acquired for the internal organs are shown in Figure 6.

In Table 5, the maximum pressure, strain, and strainrate acquired by the left lung, liver and stomach sensors are reported. The data show that the internal organ which experiences higher solicitations is the liver, followed by the left lung and finally the stomach.

**TABLE 5:** Results in terms of peak pressure, maximum strain, and maximum strainrate acquired by the left lung, liver, and stomach sensors during the thorax frontal impact simulation.

	Peak pressure [kPa]	Maximum strain [−]	Maximum strainrate [s^−1^]
Left lung	150	0.435	171
Liver	328	0.685	398
Stomach	45	0.438	242

### 4.3 Free Air Explosion of 2.3 kg C‐4 Charge

A simulation of a 2.3 kg C‐4 charge detonating at 2.34 m from a THUMS model and 1.27 m above ground was performed. In LS‐Dyna a LBE routine to take into account the ground impingement of an explosive charge is implemented, which is able to determine the entity of a reflected air blast for simple geometries such as the one proposed, hence the mentioned algorithm was used. Since the LBE method needs the TNT equivalent mass, a coefficient of 1.19 was applied for the conversion [58], resulting in 2.7 kg of equivalent TNT.

**FIGURE 7:**
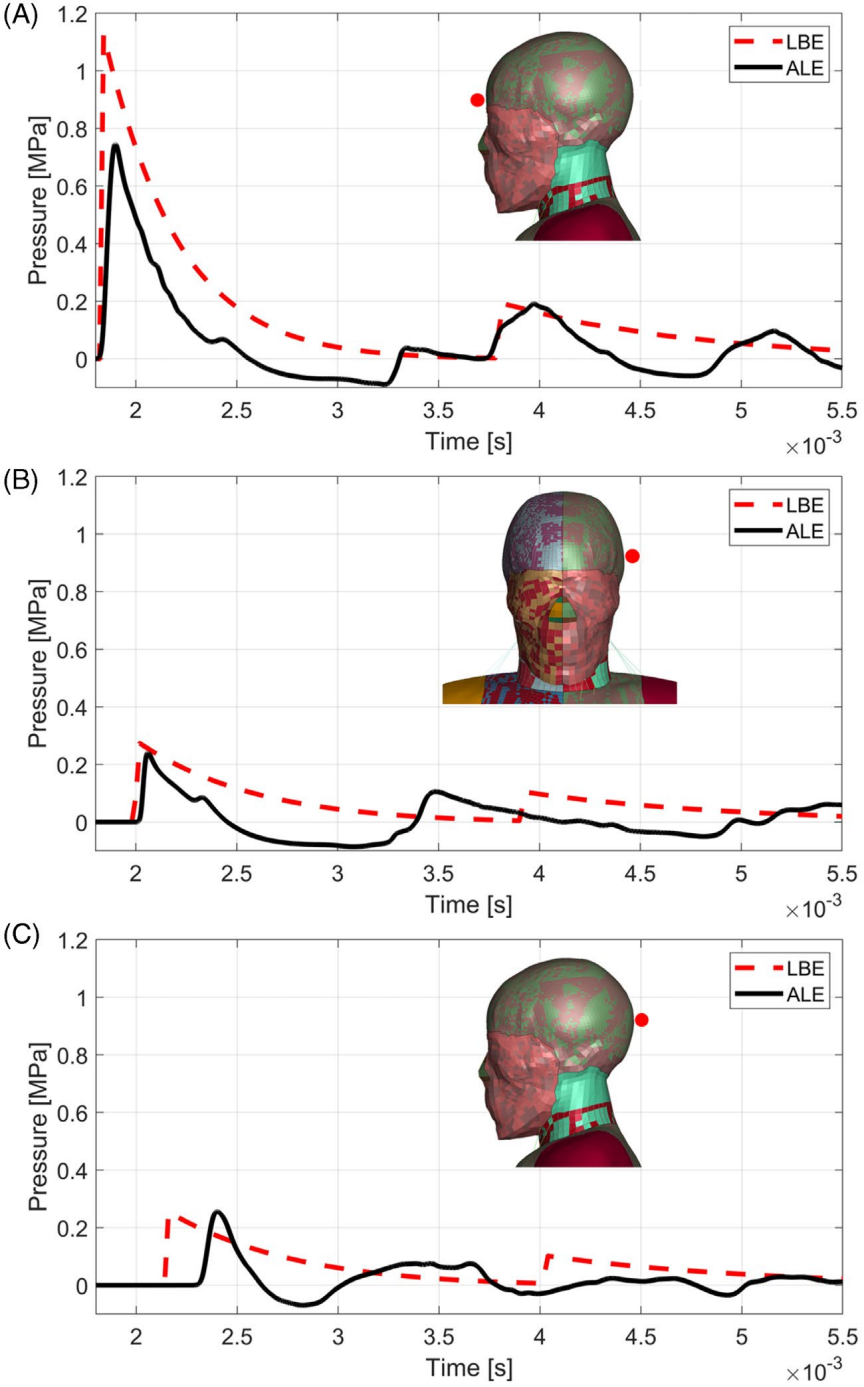
Forehead (A), temple (B) and rear (C) external head pressure gauge sensors from LBE and ALE simulations.

Pressure gauge sensors were positioned in the air at forehead, temple and rear locations to measure the blast load experienced by the human model head. In Figure 7, the pressure histories measured by the pressure gauges are reported for both the LBE and the ALE simulations. The pressure histories predicted by the LBE method with ground reflection are higher than the ALE results. The first peak can be attributed to the incident wave, while the second peak appearing at around 3.5 ms is due to the arrival of the reflected wave from the ground.

**FIGURE 8:**
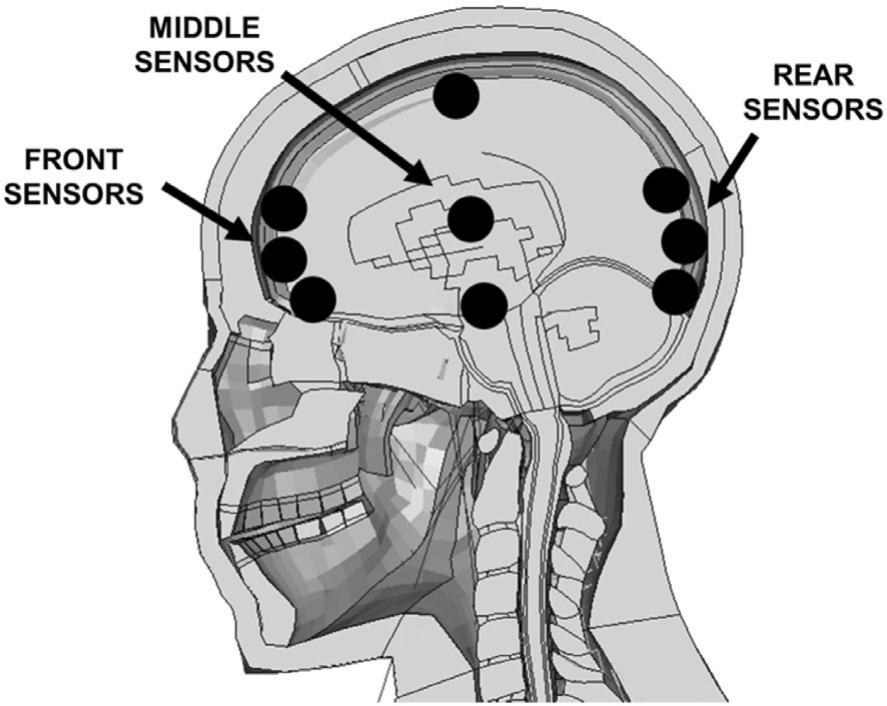
Brain front, middle, and rear sensors positions.

In order to define the solicitation imposed on the human head model, some elements of the grey matter part, which are located in the sagittal plane, were taken as numerical sensors. The elements considered are highlighted in Figure 8. In order to avoid numerical singularity errors, more than one sensor element was taken for front, middle and rear brain, however the data from sensors were averaged to obtain only one curve for each portion of the brain.

Since the blast loading conditions are similar to [47], a comparison between the results of the Zygote model specifically designed for human blast injury studies and the pedestrian THUMS model can be proposed. The human body model with anatomy by the Zygote Media Group, Inc. features an average element size of 2.5 mm, displaying a total element number of 4.2 million [47]. On the other side the THUMS v4.02 pedestrian model has an average size of around 5 mm, with elements in the head ranging from 1.2 to 5 mm, resulting in a total number of elements of 2 million [33].

Concerning the material models of the head, both in the THUMS and Zygote models the brain is modeled with a viscoelastic model with only slight differences in the coefficient values between the two models. On the other side the CSF is described by a water‐like material in [47], following the findings of Goeller et al. [15], while, in the THUMS, CSF is modeled as a viscoelastic material but with different coefficients with respect to the brain. The wider discrepancies between the two head models concern the skull, which is modeled as elastic in the Zygote model, while in the THUMS it has a material damage model. Unfortunately, no insights are given concerning internal organs material models concerning the Zygote model in [47].

**FIGURE 9:**
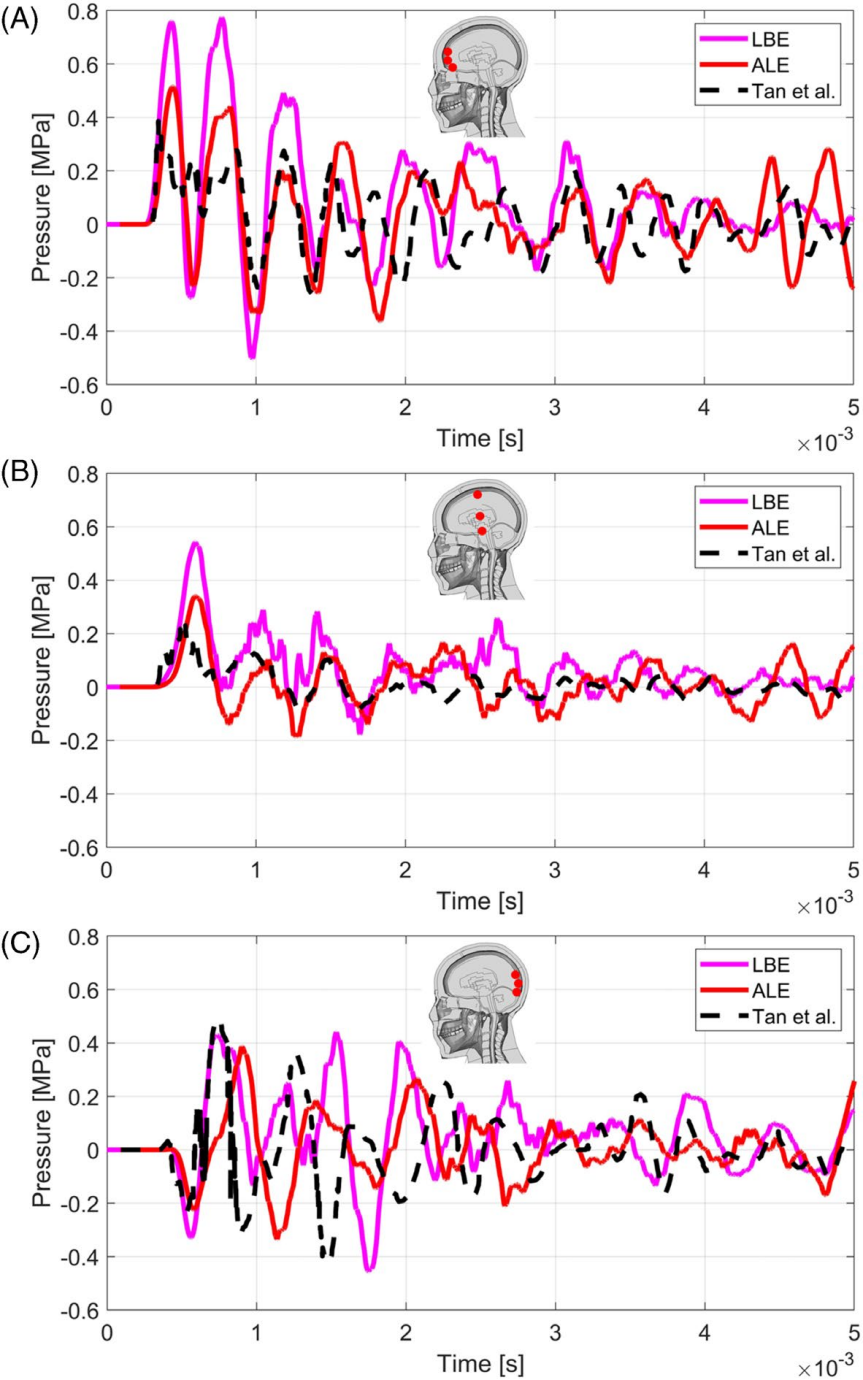
Comparison between results from numerical simulations with LBE and ALE from present work and simulation from Tan et al. [47], concerning the frontal part of the grey matter (A), the middle part (B), and the rear part (C). The time was offset by 1.6 ms with respect to the detonation.

In Figure 9 the pressure histories of the sensors in the frontal, middle, and rear part of the grey matter from the numerical simulation with LBE and ALE Coupled methods are reported alongside the results from [47]. From the comparison it is clear that the LBE technique predicts the highest pressures reached, hence depicting the most critical condition for the human brain. Indeed, ALE method from the present work shows results which are more in agreement with the simulations from [47]. It is clear that, given the differences between the THUMS model and the Zygote model both in terms of biomechanical morphology and of material models, the pressure oscillation frequencies and the peak values of the sinusoids are in good agreement.

In Table 6 the peak pressures, maximum strain, and strainrate measured by the virtual sensors in the front, middle, and rear parts of the grey matter are reported. The peak pressures are higher than those predicted by the same sensors for the frontal blunt impact (Table 4). However, the material model for the grey matter of the THUMS model is a viscoelastic model, meaning that the mechanical properties of grey matter are not influenced by the pressure level. Besides, the viscoelastic model is dependent on the rate of application of the load, hence the strainrate applied should be considered. In this sense the strainrates reached by the grey matter during the blast load are only slightly higher than those reached by the validation blunt impact case, thus the material models are extrapolating of a neglectable quantity.

**TABLE 6:** Results in terms of peak pressure, maximum strain, and maximum strainrate acquired by the front, middle, and rear sensors during the 2.3 kg C‐4 blast simulation.

	Peak pressure [kPa]	Maximum strain [−]	Maximum strainrate [s^−1^]
Front	515	0.031	19
Middle	303	0.041	31
Rear	386	0.029	33

In [59], a pressure threshold of ±0.2 MPa for brain contusion was proposed. In this sense the results reported in Figure 9 describe a situation in which the brain suffers from a severe contusion in the coup, contrecoup, and also in the middle part of the grey matter.

**FIGURE 10:**
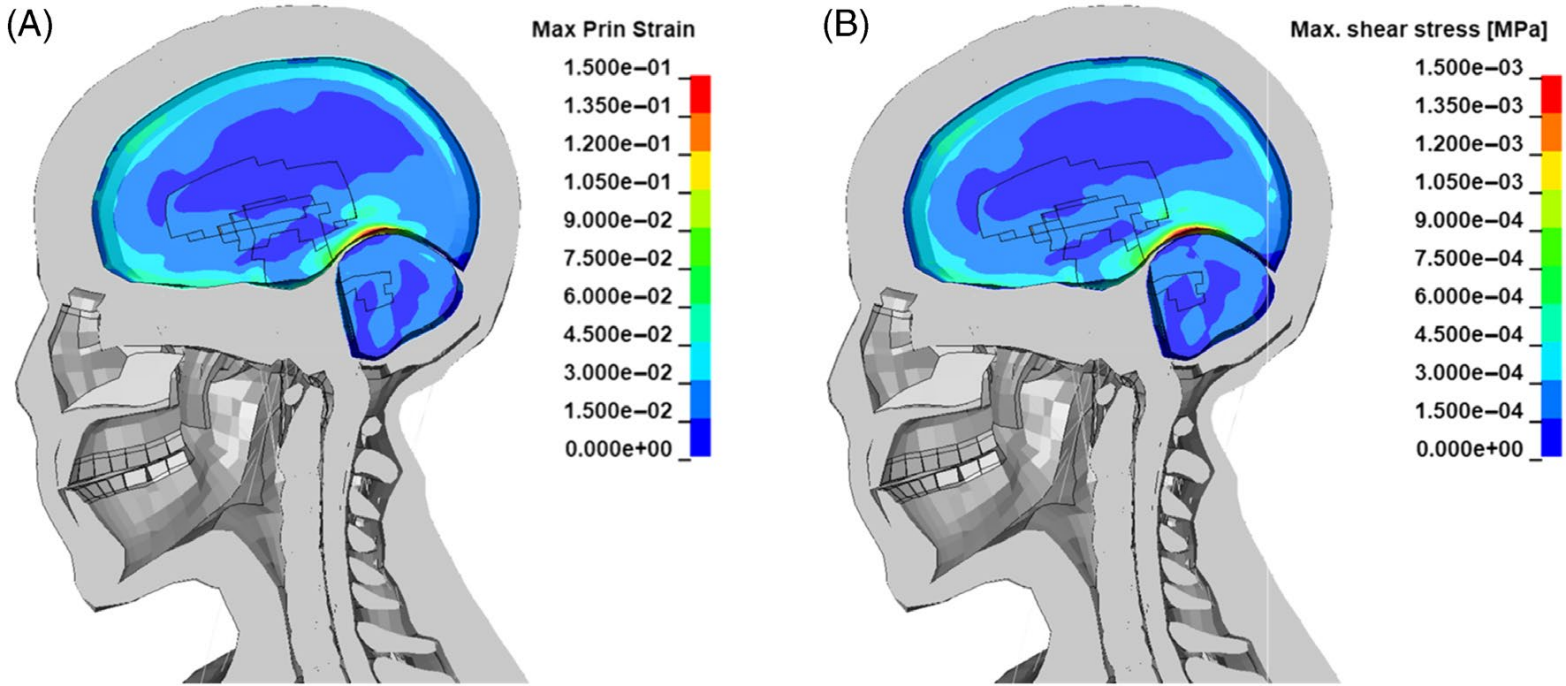
Fringe plots of grey matter and cerebellum at 7 ms from detonation of the (A) maximum shear stress (B) maximum principal strain.

More recently Zhang et al. [60] proposed a shear stress threshold for mild traumatic brain injury of 4–5 kPa. Concerning diffuse axon injury (DAI) Takhounts et al. [61] indicated a strain range of 0.15–0.25 as a threshold. At 7 ms from the detonation, a few elements of the grey matter sustained strains higher than 0.15 and shear stresses around 1.5 kPa. The portion of the brain affected by DAI according to Takhounts et al. threshold is located in the occipital lobe at the interface with the cerebellum (Figure 10a). However, shear stresses did not exceed the threshold proposed by Zhang et al. (Figure 10b).

### 4.4 Free Air Explosion of 0.9 kg C‐4 Charge

The second blast scenario generated by a 0.9 kg C‐4 charge will be used to verify the lung, liver, and stomach biomechanical models of the THUMS. Merkle et al. developed a surrogate only‐torso mannequin and exposed it to different blast conditions to evaluate its ability to acquire pressure signals from the different abdominal vital organs [16].

**FIGURE 11:**
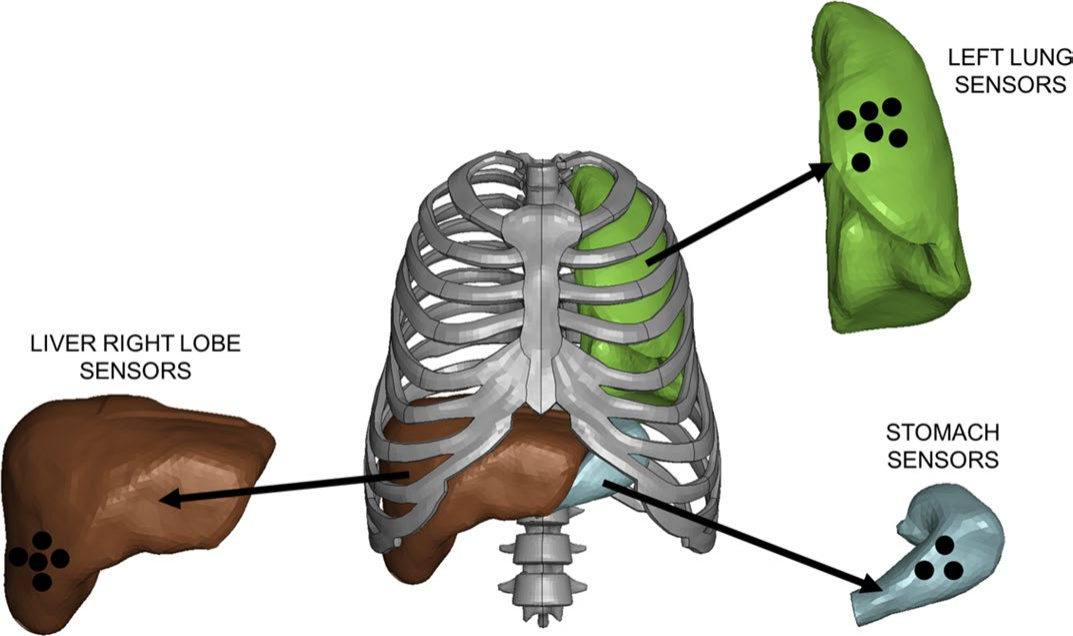
Representation of the virtual sensors positions on the THUMS applied to the left lung, left lobe of the liver and stomach.

In Figure 11, the virtual sensors locations on the pedestrian THUMS model are represented. More than one sensor was acquired for each organ to avoid numerical singularities affecting the results, successively data were averaged.

**FIGURE 12:**
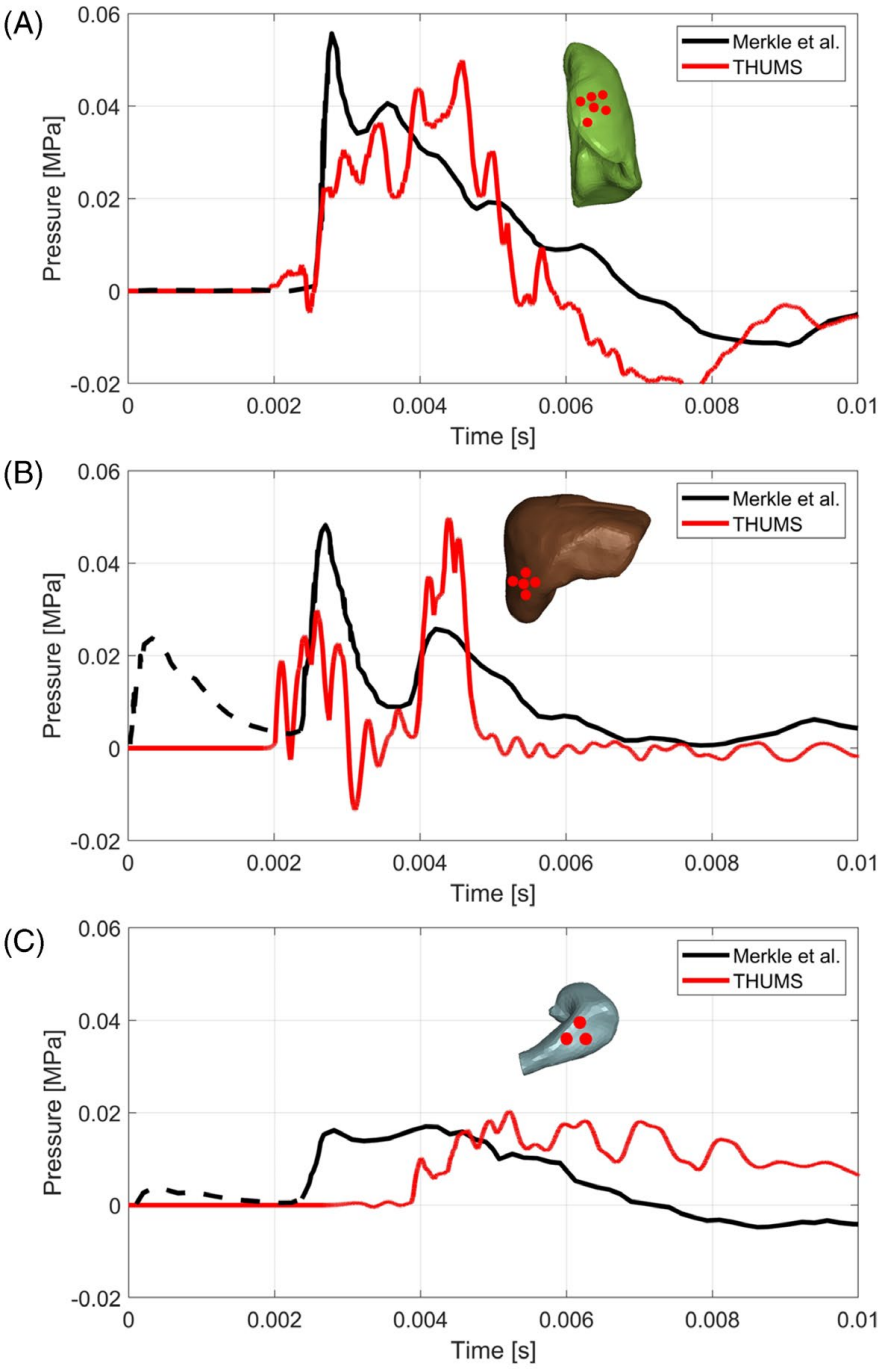
Pressure histories from [16] and simulation with THUMS pedestrian model acquired from left lung (A), liver (B), and stomach (C). The dashed part of the experimental data is the region in which an extraneous signal was detected.

The data acquired by the THUMS internal organs sensors are compared to the pressure sensors from [16] in Figure 12. As it appears evident the pressure histories are in good agreement, showing some major differences for the timing of stomach pressure sensors as the experimental sensor seems to anticipate the pressure wave arrival with respect to the simulation. Furthermore, the first spike due to the incident wave in the liver seems to be underestimated in the simulation, while the second spike due to the reflected wave from the ground is overestimated. Nevertheless the peak pressures reached by all the modeled organs, as well as pressure rising and fall times, considered are in accordance with experimental evidence.

Experimental first peak acquired both by the liver and the stomach experimental sensors was due to an extraneous signal following the charge detonation as declared by the authors [16].

The maximum pressures, strains, and strainrates experienced by the various organs investigated are summarized in Table 7. From the comparison with the data from the validation set recalled in Table 5 it appears that the material models used for lung, liver and stomach are used within the limits in which the parameters were calibrated, except for the strainrate of the lung which slightly exceeds the strainrate reached during thorax blunt impact.

**TABLE 7:** Results in terms of peak pressure, maximum strain, and maximum strainrate acquired by the left lung, liver and stomach sensors during blast simulation.

	Peak pressure [kPa]	Maximum strain [−]	Maximum strainrate [s^−1^]
Left lung	55	0.421	258
Liver	50	0.075	155
Stomach	20	0.139	118

Among the considered thoracic and abdominal organs, the lungs are those with the lower injury thresholds, and their damage could be life threatening. For this reason, in this work, lungs' blast trauma will be discussed.

Injury thresholds for lungs are often related to the peak pressure reached [62, 63, 64]. Schaffer et al. indicated a pressure threshold for lung contusion around 10 kPa for automotive crashworthiness impacts [62]. Later, Josephson and Tomlinson proposed a pressure of 240 kPa for damaging effects to the lungs during blasts [63]. Similarly, Stuhmiller et al. [64] showed that the initiation of lung injury can be established for pressures between 70 and 110 kPa for blast loadings.

The 0.9 kg of C‐4 detonation of the considered scenario is below the threshold for lung injury indicated by Bowen's curves [9], and, consistently, no element of the lungs exceeded 70 kPa of pressure.

### 4.5 Partially Confined Explosion of 2.3 kg of C‐4

The confinement due to structures surfaces generates reflections of the blast wave, hence increasing the damaging effects to the surrounding objects and people. For this reason, in this section a numerical investigation on how the presence of a wall behind a living person increases the produced injury is performed resorting to the THUMS human model. The surface behind the human model could be the facade of a building or else, however the partial confinement given by a wall placed behind has a great effect.

Thus, the 2.3 kg of C‐4 blast scenario considered previously was simulated with the addition of a rigid wall right behind the THUMS model.

**FIGURE 13:**
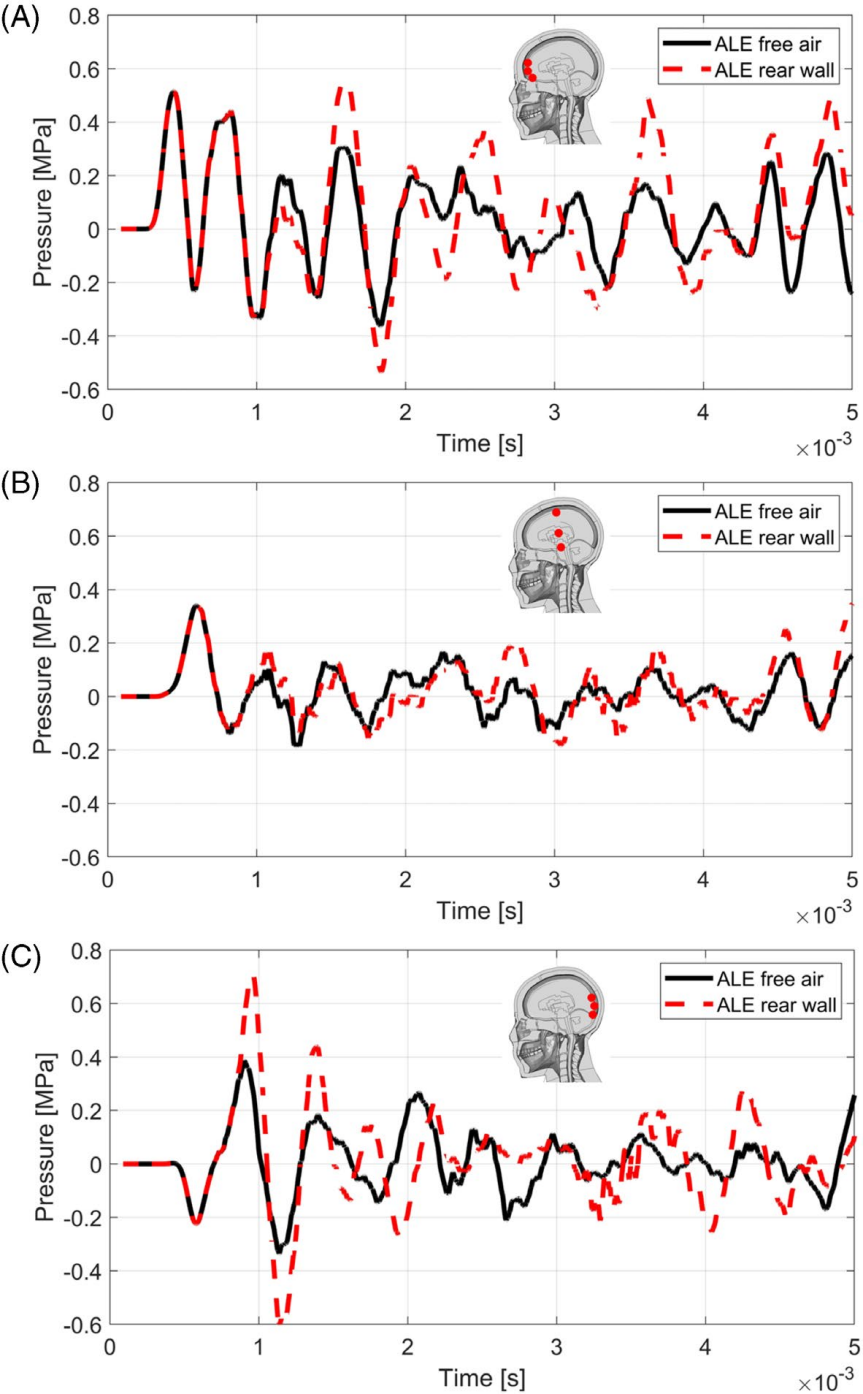
Pressure histories of the free air blast and partially confined blast ALE simulation cases, concerning the frontal part of the grey matter (A), the middle part (B) and the rear part (C). The time was offset by 1.6 ms with respect to the detonation.

The brain pressure histories are reported in Figure 13 for the free air and the rear wall confined simulation cases. As it appears evident, the rear wall confinement causes an increase in pressure levels of the contrecoup part of the brain with a positive pressure peak which exceeds 0.6 MPa, which is three times higher than the brain contusion threshold proposed by Ward et al. [59]. The blast wave reflected from the rear wall causes an increase of around 0.25 MPa of the first positive pressure peak in the rear portion of the brain. Indeed, also the coup experiences higher stresses given both by the waves travelling inside the grey matter and by the reflected blast.

Surprisingly the middle part of the brain seems not to be greatly influenced by the rear wall confinement with only slight differences in the values of the peaks.

**FIGURE 14:**
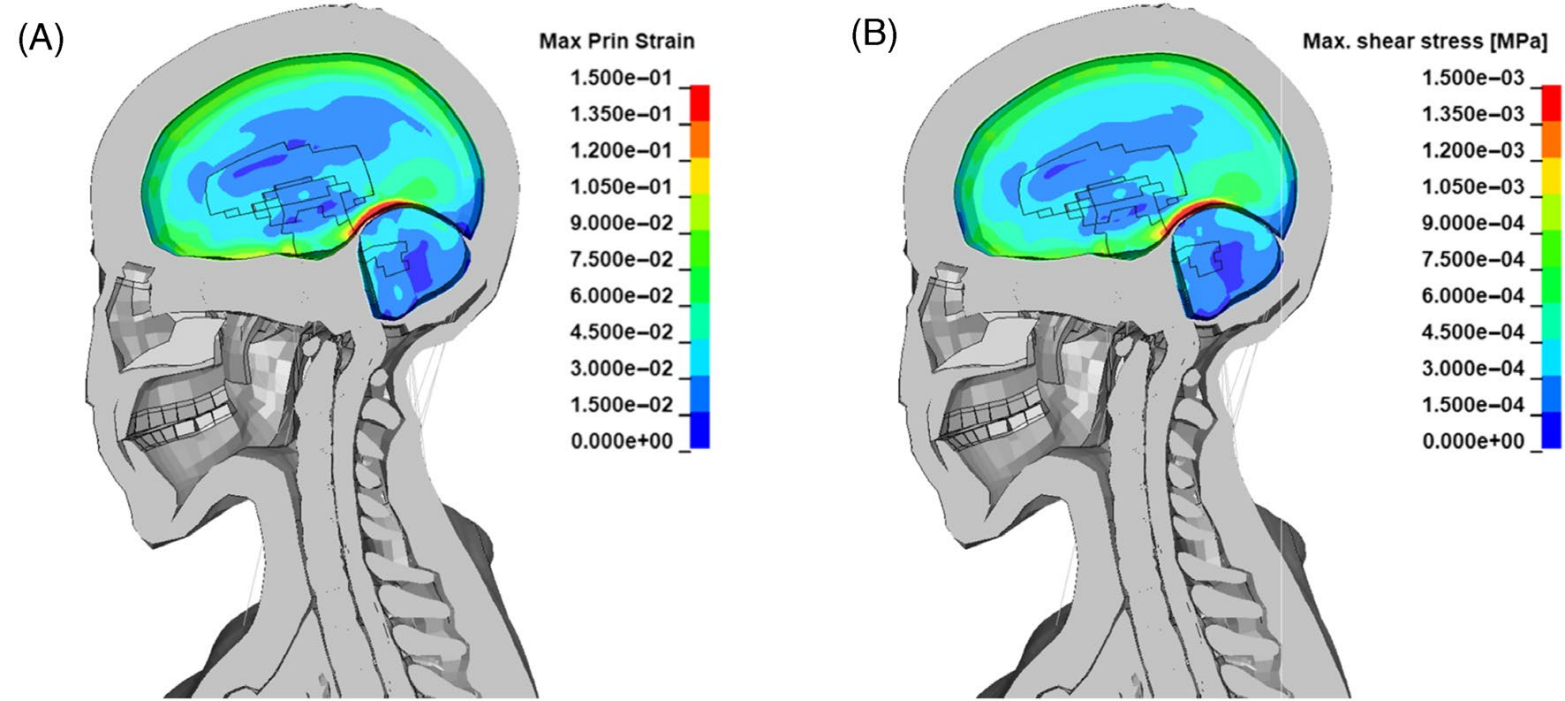
Fringe plots of grey matter and cerebellum at 9 ms from detonation of the (A) maximum shear stress (B) maximum principal strain.

In the partially confined case, the peak of maximum principal strain and of maximum shear stress is reached at 9 ms from the detonation (Figure 14). The entity of the strains is such that DAI might occur in a wider portion of the grey matter with respect to the free blast scenario, according to Takhounts et al. threshold [61]. Similarly the shear stresses are higher, with a peak of 2 kPa in the occipital lobe, but still within the limits proposed by Zhang et al. for brain contusion [60].

Interestingly, the head of the human model moves more in the posterior direction (i.e., from coup to contrecoup) in the rear wall case with respect to the free air case. However, during the 12 ms of simulation time the head did not hit the wall in both the simulated cases, hence no estimation of the tertiary injuries was made.

**FIGURE 15:**
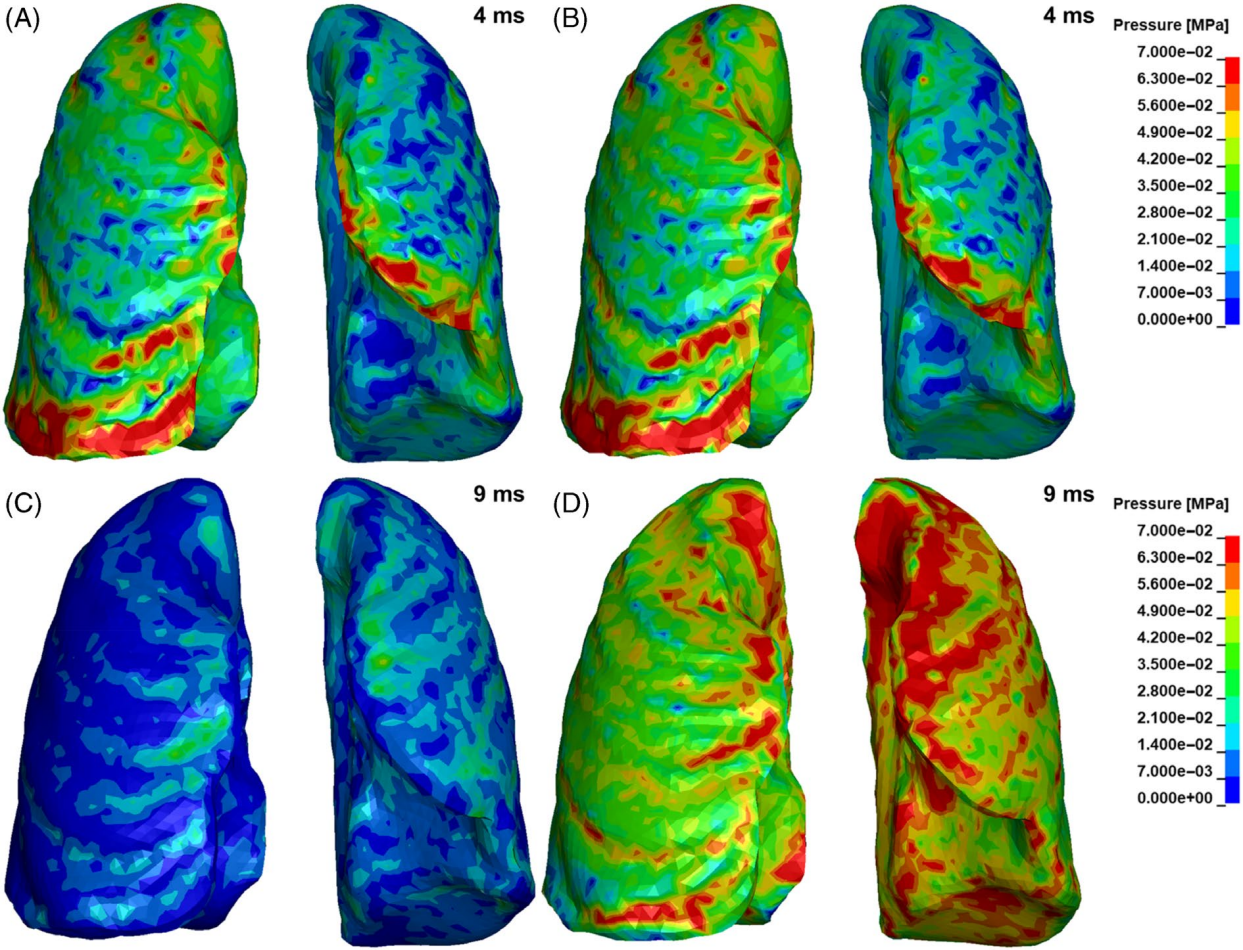
Pressure fringe plots of the lungs for the free air blast at 4 ms (A), at 9 ms (C), and for the partially confined blast at 4 ms (B), at 9 ms (D).

Concerning lung trauma, in Figure 15 the pressure fringe plots of the lungs for the free air and partially confined blast are reported at 4 and 9 ms from the detonation. From the comparison of the pressure fringe plots of the lungs at 4 ms for the free air and partially confined scenario it is clear that the two simulation are describing a similar condition with few elements exceeding the injury threshold of 70–110 kPa indicated by [64]. As a matter of fact at 4 ms no reflection of the blast wave has happened yet, hence there are no differences between free air and partially confined explosion setups.

On the other hand, the comparison at 9 ms from the detonation shows a more critical condition, particularly for the left lung. In fact, a wide area of the left lung exceeds the lung injury criterion of 70 kPa (Figure 15d).

**FIGURE 16:**
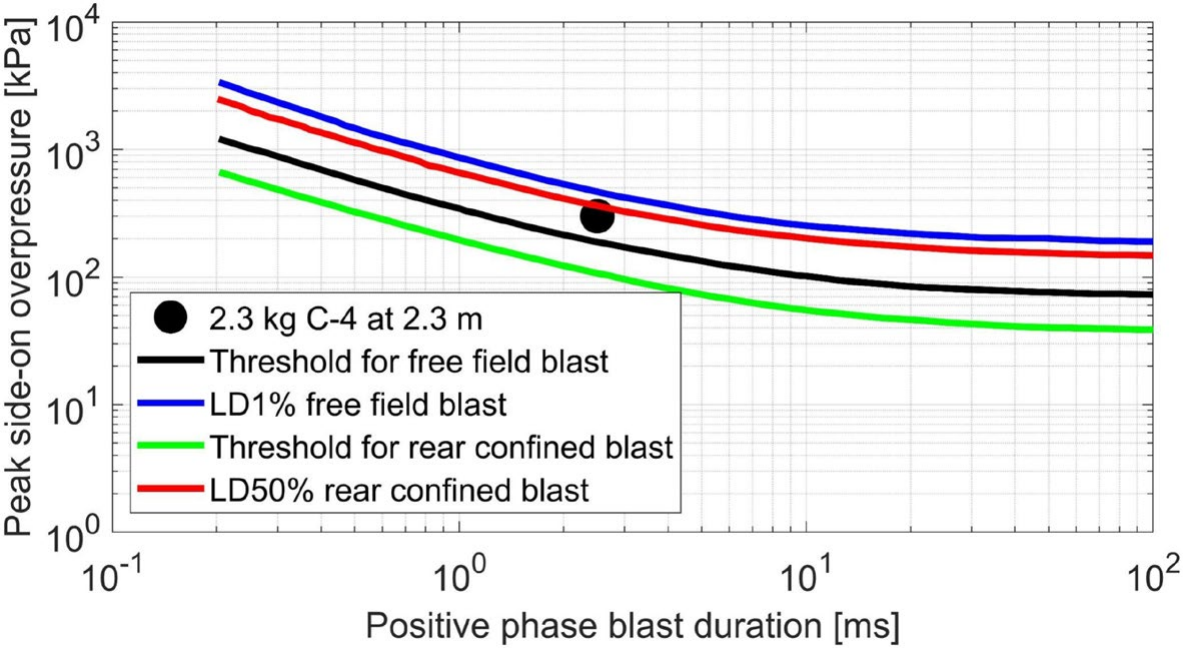
Plot of the Bowen curves for lung injury [9] and comparison with the simulated blast of 2.3 kg of C‐4 composition at 2.3 m of distance.

The increase in lung trauma caused by the rear wall confinement shown by the simulations of the present work could be corroborated by the comparison with the well‐known Bowen's curves (Figure 16). In spite of the age of the research by Bowen et al. [9], Bowen's threshold curves are still taken in great consideration by many authors [1, 18, 65].

Indeed, the 2.3 kg of C‐4 detonation describes a condition which is slightly more dangerous than the threshold for lung injury for a free field blast, and this statement finds evidence in the presence of some regions of the lungs which experienced a pressure higher than 70 kPa (Figure 15a). However, the confinement due to a reflecting surface lowers the Bowen's curves, hence the rear confined blast scenario describes a condition near to 50% survival probability related to lung trauma. Thus, the wider region of the lungs subjected to more than 70 kPa depicted by THUMS partially confined blast simulation (Figure 15d) is in agreement with Bowen's findings.

## 5 Conclusions

The present work concerns the investigation of the THUMS pedestrian human model as a tool to predict blast‐related human injuries. The THUMS model is a detailed biomechanical finite element surrogate which is being developed by the Toyota Motor Company for automotive crashworthiness simulations and it is now publicly available.

Firstly, a validation of the ALE method used throughout the study is presented. An experimental test on blast deformed steel boxed is taken into account. Thus, the authors simulated the deformation of a 450 mm wide steel box experimental case. Consequently, given that the aim of the work is to predict structures wide enough to contain people, the small steel box was scaled to have 3000 mm wide edges, and the explosive was scaled accordingly. The numerical results of the wider steel box deflection were coherent with the DIC data from the small boxes.

The validation procedure highlighted the characteristics of LBE and ALE approaches, whose knowledge is of paramount importance to develop accurate numerical simulations of human blast injuries.

After the demonstration of validity of the proposed techniques, the THUMS human model validation cases relevant for the current study were presented. The THUMS model is composed of a wide variety of material models describing the mechanical behavior of all the tissues and bones. These material models were calibrated for a certain range of pressures and strainrates. The automotive incident scenarios generate a state of solicitation on a living person which is in general different from the solicitation caused by a detonation. Thusly, it is fundamental to understand the limits of validity of the THUMS model in order to be able to check whether it could be used for the simulation of blast scenarios.

For this reason the limits in terms of pressures and strainrates of the validation cases were detailed.

In order to demonstrate the validity of the THUMS model to simulate human response to explosions, three blast scenarios were simulated. The first blast scenario is the detonation of a 2.3 kg of C‐4 composition at 2.3 m from the THUMS. The present case was chosen because it lies in the proximity of the blast lung injury for free field blasts. In the literature no experimental data on PMHS or surrogates exposed to the aforementioned scenario are present, thus a numerical investigation by [47] was taken for comparison. Tan et al. developed their own human model specifically for blast simulation and analyzed the head response to the explosion of 2.3 kg of C‐4 at 2.3 m.

From the comparison of the head pressure sensors of the THUMS simulation and of the data from [47] it appears that the Toyota human model is able to reproduce pressure waves in the grey matter consistently with a human model built purposefully for blast analysis.

The second blast scenario was generated by 0.9 kg of C‐4 at 2.3 m from the THUMS. In this case the biofidelity of THUMS thoracic and abdominal organs was investigated through the comparison with an experimental campaign by [16]. The experimental pressure histories recorded by the left lung, liver and stomach sensors during Merkle et al. tests were satisfactorily simulated by the virtual pressure sensors of the THUMS, both in terms of peak pressures and pressure rise and fall times.

Finally, the 2.3 kg of C‐4 charge was again detonated at 2.3 m from THUMS but this time the human model was standing right in front of a wall. The objective of the simulation of this blast scenario was to verify whether the proposed numerical approach is able to correctly reproduce the increase in lethality due to the partial confinement of the present setup. Hence, the grey matter and lungs response to the third blast case was compared to the first blast case. As expected, the THUMS predicted more critical conditions for both the brain and the lungs.

For all the proposed blast scenarios, some injury thresholds for brain and lungs taken from the literature are taken as reference and commented alongside the results of the simulations developed in this work. Furthermore, the limits of validity of the material models of the investigated organs are commented for each case resulting in the fact that the THUMS model has been used in the range of its calibration parameters. Probably, in harsher blast scenarios the material models could be used in too wide extrapolation, hence seriously reducing THUMS' accuracy.

In conclusion, the THUMS pedestrian human model biofidelity was analyzed for explosion cases, and its ability to simulate the response to accidental blast exposure was assessed through comparison with other human models and experimental data. The THUMS model reported results which agree with experimental data and existing injury thresholds, hence in the authors' opinion it is suitable to be used for blast scenarios similar to those investigated in the present study. Other blast scenarios will be investigated in future studies.

By using a human model publicly available, the present work aims to lay the groundwork for making the simulation of explosion effects on the human body more accessible to the public. The goal is to develop a common framework which could progress research in this field, also by using THUMS open‐source model to reproduce experimental investigations by analyzing results on different vital organs.

## Conflicts of Interest

The authors declare no conflicts of interest.

## Data Availability

The data that support the findings of this study are available from the corresponding author upon reasonable request.
